# Similar short-term efficacy of oral levosulpiride and intravitreal ranibizumab in patients with diabetic macular oedema

**DOI:** 10.1038/s41433-025-04182-6

**Published:** 2025-12-24

**Authors:** Elva Adán-Castro, Carlos D. Núñez-Amaro, Julián Villarreal, Ilse H. Islas, Marlon García-Roa, Ellery López-Star, Renata García-Franco, Ma. Ludivina Robles-Osorio, Gonzalo Martínez de la Escalera, Carmen Clapp

**Affiliations:** 1 Instituto de Neurobiología, Universidad Nacional Autónoma de México (UNAM), Campus UNAM-Juriquilla, Querétaro, México; 2 Instituto Mexicano de Oftalmología (IMO) IAP, Querétaro, México; 3 Instituto de la Retina del Bajío (INDEREB), Querétaro, México; 4 Facultad de Ciencias Naturales, Universidad Autónoma de Querétaro (UAQ), Querétaro, México

**Keywords:** Hormonal therapies, Pharmaceutics

Diabetic macular oedema (DMO) is a major cause for vision loss. Primary treatment involves intravitreal injections of inhibitors of vascular endothelial growth factor (VEGF), but suboptimal responders and the burden of frequent injections demand less invasive treatments such as oral medications [1]. Levosulpiride (LSP), a prokinetic dopamine D2-receptor blocker, was recently repositioned as an affordable and safe oral treatment for DMO [2]. Oral LSP for 8 weeks improved visual and structural outcomes in patients with centre-involving DMO [2] by different mechanisms including: (a) blockage of dopamine D-2 receptors at the pituitary level causing the hyperprolactinemia-mediated increase in intraocular vasoinhibin [3], a prolactin fragment that inhibits retinal vascular leakage induced by VEGF and diabetes; and (b) downregulation of VEGF and placental growth factor in the eye [2].

Oral medications favour compliance and offer an earlier intervention against the worsening of DMO in patients with good vision when intravitreal anti-VEGF injections are not used due to their invasiveness and high cost [4]. A key question is whether oral LSP could be as effective as intravitreal anti-VEGF for the early treatment of DMO. Here, we addressed this question by comparing the short-term benefit of LSP to that of the widely used intravitreal ranibizumab (RBZ), a humanized monoclonal anti-VEGF fragment [5].

A prospective, double-blinded, placebo-controlled, dual-centre, phase 2 trial (ClinicalTrials.gov, NCT03161652) was conducted from May 2017 to June 2025 in type 2 diabetic mestizo patients with centre-involving DMO. Protocol details were reported [2]. Eligible participants [aged 40-69 years, best corrected visual acuity (BCVA) between 58 to 14 Early Treatment Diabetic Retinopathy Study (ETDRS) letters at 4 m (20/16 to 20/125 Snellen equivalent), and central foveal thickness (CFT) > 224 µm] were randomly assigned to placebo (lactose pill TID), LSP (DISLEP, Ferrer Therapeutics, 25 mg pill TID), or placebo (TID) and RBZ (Lucentis, Novartis, 0.5 mg per month intravitreal injection) treatments initiated after signing the informed consent and during a follow-up of 8 weeks.

Data on placebo and LSP, including losses/exclusions, were reported [2] but not from placebo and RBZ, where hyperprolactinemia excluded 2 patients. The demographics, clinical, and ophthalmic characteristics of the 47 patients completing the study were similar at baseline. Hyperprolactinemia after 8 weeks confirmed adherence to LSP treatment (Table 1). LSP and RBZ met primary endpoints of non-inferior mean values in BCVA and anatomical outcomes [CFT and mean macular volume (MMV) defined by OCT] at 8 weeks. BCVA, CFT, and MMV values were comparable among groups (Table 1). Mean longitudinal changes over baseline in BCVA, CFT, and MMV were higher after LSP and RBZ relative to placebo and similar between LSP and RBZ (Fig. 1). The % of eyes improving over placebo were comparable between LSP and RBZ in BCVA (82 vs. 100%), CFT (91 vs. 94%), and MMV (80 vs. 87%).

**Fig. 1:**
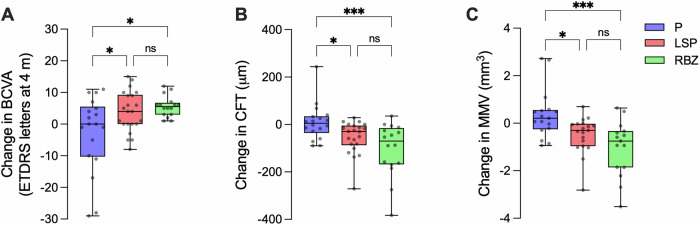
Comparable improvements by 8-week oral levosulpiride (LSP) or intravitreal ranibizumab (RBZ) on the change from baseline in visual and structural parameters. Longitudinal changes from baseline in best-corrected visual acuity (BCVA) (**A**), central foveal thickness (CFT) (**B**), and mean macular volume (MMV) (**C**) after 8 weeks of treatment. Boxplots show median and individual values distribution of placebo (P, 18 eyes), LSP (22 eyes), and RBZ (16 eyes) groups. Statistical analysis was performed using one-way ANOVA-Sidak’s multiple comparisons (**A**) or Kruskal-Wallis-Dunn’s multiple comparisons (**B**, **C**) tests. *P < 0.05, ***P < 0.001, ns (non-significant).

**Table 1:** Demographic, clinical, and ophthalmic characteristics of the diabetic macular oedema groups.

Characteristic	BEFORE TREATMENT				AFTER 8-WEEK TREATMENT			
	Placebo (17 patients 18 eyes)	LSP (17 patients 22 eyes)	RBZ (13 patients 16 eyes)	*P*	Placebo (17 patients 18 eyes)	LSP (17 patients 22 eyes)	RBZ (13 patients 16 eyes)	*P*
**Age years (SD)**	61.0 (7.3)	58.5 (6.8)	61.7 (5.8)	0.393^b^				
**Sex F n (%)**	5 (29.4)	7 (41.2)	4 (30.8)	0.737^a^				
**DM2 years (SD)**	16.9 (7.0)	19.4 (13.3)	18.1 (8.6)	0.764^c^				
**HbA1c (SD)**	8.8 (1.5)	8.1 (1.8)	7.3 (1.5)	0.063^c^				
**SPRL ng/mL (SD)**	7.3 (2.4)	8.6 (3.6)	7.9 (3.2)	0.482^b^	7.7 (3.5)	150.1 (112.2)	9.8 (6.5)	<0.0001^c^
**BCVA ETDRS lletters (SD)**	37.3 (10.8)	36.4 (8.7)	35.44 (10.2)	0.855^b^	34.9 (15.1)	39 (11.4)	41 (10.5)	0.350^b^
**CFT µm (SD)**	325.9 (59.9)	374.2 (89.5)	395.1 (108.0)	0.062^c^	336.8 (70.5)	342.6 (105.1)	292 (92.8)	0.078^c^
**MMV mm**^**3**^ **(SD)**	8.5 (0.9)	8.5 (1.2)	9.1 (1.2)	0.294^c^	8.9 (1.1)	8.4 (1.5)	8.1 (0.9)	0.040^c^

Values represent means of the number of patients (first 5 rows) or eyes (last 3 rows). *LSP* levosulpiride, *RBZ* ranibizumab, *SD* standard deviation, *F* female, *DM2* type 2 diabetes mellitus, *HbA1c* glycosylated haemoglobin; *SPRL*, serum prolactin, *BCVA* best-corrected visual acuity; *ETDRS* Early Treatment Diabetic Retinopathy Study letters at 4 m, *CFT* central foveal thickness, *MMV* mean macular volume. Intergroup differences (*P*-values) were analysed by chi-square test^a^, one-way ANOVA^b^ or Kruskal–Wallis test^c^.

Non-inferior gains in visual and structural outcomes relative to RBZ support LSP as an effective, non-invasive alternative for the early intervention of DMO. Strengths include randomization, placebo use, double-blinded nature, similar baseline values, and no adverse-side effects. Larger/longer studies await further research.

## Data Availability

All original data generated or analysed during the current study are included in this published article.
